# Effects of postharvest treatments with chitosan and putrescine to maintain quality and extend shelf‐life of two banana cultivars

**DOI:** 10.1002/fsn3.662

**Published:** 2018-05-29

**Authors:** Marjan Sadat Hosseini, Seyed Morteza Zahedi, Javier Abadía, Mahdieh Karimi

**Affiliations:** ^1^ Institute for Agriculture Biotechnology Research – Isfahan Branch Agricultural Research, Education and Extension Organization (AREEO) Isfahan Iran; ^2^ Department of Plant Nutrition Aula Dei Experimental Station (CSIC) Zaragoza Spain; ^3^ Department of Horticultural Science Faculty of Agriculture University of Maragheh 551181‐8311 Maragheh Iran; ^4^ Department of Horticultural Sciences Bu‐Ali Sina University Hamedan Iran

**Keywords:** antioxidant activity, chitosan, ethylene, microbial contamination, polygalacturonase, putrescine

## Abstract

Chitosan (1.0% and 2.0%) and putrescine (1.0 and 2.0 mmol/L) treatments were used to investigate the effects of these compounds on the postharvest quality and shelf‐life of two banana cultivars, “Native” and “Cavendish.” Fruits were stored at 15 ± 2°C and a relative humidity of 85%–90% during a 20‐day period. In the controls, increases in weight loss, microbial population, total soluble solids, and ethylene production and decreases in firmness, ascorbic acid contents, and fruit lightness occurred gradually during storage. All these changes were delayed significantly after treatments with chitosan and putrescine. Application of putrescine and chitosan also caused small increases in phenolic compound contents and antioxidant activity at the end of the storage period. Results obtained suggest that a treatment with 1% chitosan is effective in improving the postharvest quality and shelf‐life of banana, and open the possibility that lower concentrations of chitosan may be also effective.

## 1 INTRODUCTION

Banana (*Musa* spp.) is one of the most popular and important commercial fruits worldwide. It is native to tropical areas of the world, and its global production reached 144 million t in 2014 (FAO, 2014). Regarding nutritional values, banana is rich in minerals, vitamins, carotenoids, and other antioxidants, and apart from being consumed fresh, it is often used as a flavoring and coloring agent in food industries. Due to the limited firmness of the tissue, fully ripe banana fruits are sensitive to physical damages during storage and transportation, and therefore, they are harvested in green ripe stage or in early stages of the color change from green to other colors, depending on the cultivar. As banana is a climacteric fruit and has a lot of nutrients, it is prone to rotting when microorganisms are present (Malmiri, Osman, Tan, & Rahman, 2011). The postharvest shelf‐life of the climacteric fruits can be extended by preventing ethylene increases. Therefore, a variety of commercial methods involving controlled atmospheres have been applied to decrease the amount of exogenous ethylene (Wang & Qi, 1997), whereas preventing production of fruit endogenous ethylene requires characterization and application of special natural substances and coverings that do not pose a risk for human health.

The application of natural and edible coverings is an important part of the postharvest research area, aimed at controlling fruit ripening and putrescence (Romanazzi, Lichter, Mlikota Gabler, & Smilanick, 2012). Chitosan, a natural polysaccharide, creates a membrane permeable to gases such as O_2_ and CO_2_, and has been reported to maintain the quality, health, and physical properties of the fruits. This natural layer allows only a certain amount of gases to enter and exit fruits, and therefore prevents anaerobic respiration and putrescence (Lin et al., 2011). Application of chitosan in mango (Chien, Sheu, & Yang, 2007) and banana (Sakif, Dobriansky, Russell, & Islam, 2016; Suseno, Savitri, Sapei, & Padmawijaya, 2014; Zahoorullah, Dakshayani, Rani, & Venkateswerlu, 2017) has been shown to improve fruit quality, maintain firmness, reduce ethylene production and fungal contamination, delay ripening and senescence, and reduce color changes. Polyamines are a well‐known group of compounds in animals, plants, and microorganisms, and, due to their aliphatic nitrogen structure, are also among biomaterials that can help to control fruit ripening. In mango, application of polyamines increased shelf‐life, vitamin C content, and fruit color (Malik & Singh, 2005) and increased phenolics (Razzaq, Khan, Malik, Shahid, & Ullah, 2014). Polyamines have also been reported to decrease weight loss and increase firmness in pomegranate (Mirdehghan et al., 2007), and decrease ACC oxidase activity in tomatoes (Li, Parsons, Liu, & Mattoo, 2005) and fungal contamination in grapes (Mirdehghan and Rahimi, 2016).

Only a few studies have focused on the postharvest application of chitosan and putrescine to decrease mechanical damages and putrescence in banana (Purwoko, Susanto, & Novita, 2002; Sakif et al., 2016; Suseno et al., 2014; Widodo, Zulferiyenni Ginting, Fazri, & Saputra, 2015; Zahoorullah et al., 2017). Therefore, the aim of this research was to investigate the possible effects of postharvest application of different concentrations of these two edible coverings, chitosan and putrescine, to maintain the quality and increase the shelf‐life of two banana cultivars.

## 2 MATERIALS AND METHODS

### 2.1 Materials

Fruits of two cultivars of banana (*Musa* spp.), “Native” and “Cavendish,” were obtained from a commercial garden in Minab (Hormozgan Province, Iran; 27°07′N; 57°05′E) at their commercial maturity (ripe green) stage and immediately transferred to the laboratory. The cultivars were of high quality and with a relatively good resistance to rotting. Fruits selected were healthy and had no physical damages and fungal contamination, and were uniform in shape, size, color, and ripening stage. Fruits were randomly divided into eleven groups. After being washed with distilled water and air‐dried, their initial physical and chemical properties were measured (day zero). Fruits were then treated with distilled water (control), chitosan at 1% (Chi 1%), chitosan at 2% (Chi 2%), putrescine at 1.0 mmol/L (Put 1 mmol/L), or putrescine at 2.0 mmol/L (Put 2 mmol/L). Chitosan (>93% purity, CAS number 9012‐76‐4; Sigma‐Aldrich, Taufkirchen, Germany) and putrescine (putrescine hydrochloride ≥98% purity, CAS number 333‐93‐7; Sigma‐Aldrich) solutions were prepared in 0.5% acetic acid and distilled water, respectively. Then, fruits were stored at 15 ± 2°C and 85%–90% relative humidity during a 20‐day period. The measurements included weight loss, firmness, microbial analysis, lightness, total soluble solids (TSS), titratable acidity (TA), total antioxidants, total phenolic compound contents, ascorbic acid contents, polygalacturonase (PG) activity, and ethylene. All measurements were carried out on days 0, 10, and 20 after the start of the experiment, and three replications were used for all parameters measured.

### 2.2 Weight loss and firmness

Banana fruits were weighed with a digital scale (0.01 g precision) at the beginning of the experiment (day 0) and at 10 and 20 days during storage. The percentage of weight loss was calculated as: (weight‐initial weight)/(initial weight) × 100.

To determine firmness, a penetrometer device (OSK‐I‐10576; Ogawa Seiki Co., Tokyo, Japan) was used with unpeeled fruits. The mean pressure (as *N*) was recorded at three points in the fruits, in the middle, proximal, and distal parts.

### 2.3 Microbial population analysis

To measure microbial population, 90 ml of sterile peptone water (Oxoid, Basingstoke, UK) was mixed with 10 g of peeled fruit tissue in a stomacher blender device (Bag Mixer 400; InterScience, St.‐Nom‐La‐Bretèche, France). The blend was diluted 10‐fold, and 1 ml of sample was added to an agar plate (Mold Count Plates, ABRI, Karaj, Iran) to determine mold counts. Plates with 30 to 300 colonies were used for the measurements. Plates were incubated at 25°C for 5 days, and microbial population was calculated as the logarithm of the number of colonies per 10 g of fruit fresh weight (Valverde et al., 2005). Three replicates per sample were measured.

### 2.4 Lightness, TSS, and TA

Lightness changes were measured on two sides of the fruits using a colorimeter (CR 400; Konica Minolta, Osaka, Japan). The L* values were recorded and reported as zero for black (full darkness) and 100 for white (full brightness) (Ali, Mahmud, Kamaruzaman, & Yasmeen, 2011). TSS in fruit juice (obtained by homogenizing 30 g of peeled fruit tissue with 90 ml of distilled water for 2 min) were measured in Brix degrees at 25°C using a digital refractometer (A.PAL‐1; ATAGO, Tokyo, Japan). Titratable acidity in fruit juice was measured by diluting 10 ml of juice with the same volume of distilled water and then titrating with 0.2 N NaOH until the pH of the solution reached 8.4. The TA percentage was calculated according to Roussos, Sefferou, Denaxa, Santili, and Stathis (2011).

### 2.5 Total antioxidant activity, total phenolic compounds, and ascorbic acid (vitamin C) content

Tissue was collected from different parts of the fruit, frozen in liquid N_2,_ and 5 g of tissue was homogenized in 10 ml of 50 mmol/L phosphate buffer at pH 7.8. Then, the homogenate was centrifuged at 15000 × *g* at 4°C for 20 min and the supernatant (fruit extract) was used for analysis of total antioxidant activity, phenolic compounds, and ascorbic acid.

Total antioxidant activity was assessed using the 2,2‐diphenyl‐1‐picrylhydrazyl (DPPH) assay. Fifty μl of fruit extract was added to 1.0 ml of 60 μmol/L DPPH (free radical, 95%; Sigma‐Aldrich Chemie GmbH, Steinheim, Germany) in methanol. After being shaken, the mixture was left at room temperature for 30 min, and then, absorbance at 515 nm was measured with a spectrophotometer (Cary 100; Richmond, VA, USA). Methanol was used as a control (Dokhanieh, Aghdam, Aghdam, & Hassanpour, 2013).

For phenolic compound analysis, 100 μl of fruit extract was mixed with 400 μl phosphate buffer and 2.5 ml of Folin reagent (Sigma‐Aldrich). After 1 min, 2 ml of Na_2_CO_3_ (7.5%) was added to the mixture and the sample kept at 50°C for 5 min, before measuring the absorbance at 760 nm with a spectrophotometer (Cary 100). Gallic acid was used as a standard, and results were expressed as mg of gallic acid per 100 g of fresh weight (FW) (Mirdehghan and Rahimi, 2016).

The ascorbic acid concentration in fruit extracts was determined by titration using a solution containing I and KI (16 g KI and 1.72 g I in 1 L water). The titration ended when the sample turned dark blue and color was stable for a few s. The volume of the I+KI solution was recorded and the concentration of ascorbic acid calculated according to the following equation (O’Grady, Sigge, Caleb, & Opara, 2014) as ([0.88 × *V*]/5 × 100), where *V* is the volume of the consumed I+KI solution.

### 2.6 PG enzyme activity

The PG activity was measured with the method of Guo et al. (2014). After extraction of 1 g of fruit tissue with 2 ml of cold Na acetate buffer (pH 6.0) and centrifugation at 15000 × *g*, 0.1 ml of the supernatant was mixed with 0.3 ml of 0.1% (w/v) polygalacturonic acid (Sigma‐Aldrich) in 0.2 ml of 40 mmol/L Na acetate and 0.4 ml of water, and incubated at 37°C for 60 min. Then, the vessel containing the sample was immersed in boiling water for 5 min, cooled on ice, and 10 ml of distilled water was added. The absorbance at 540 nm was determined with a spectrophotometer (Cary 100).

### 2.7 Ethylene production

One hundred gram of fruit fresh weight was collected and placed in a 500 ml glass vessel at 25°C for 1 hr. Then, 1 ml of the air from above the fruit sample was collected and injected into a gas chromatography device to measure the produced ethylene (Shimadzu, Japan). The device was equipped with a flame ionization detector, and determination was carried out at 80°C with N_2_ as carrier gas (Sayyari, Soleimani Aghdam, Salehi, & Ghanbari, 2016). Values measured were reported in ng ethylene kg^−1^ fruit s^−1^.

### 2.8 Statistical analysis

The analyses included different treatments, three storage times and two banana cultivars. Data were analyzed using a factorial completely randomized design with three replications. Statistical analysis of the experimental data was conducted by a general linear method (GLM) using the SAS software (v. 9; SAS 2003), and the mean comparison was conducted using the multidomain Duncan test at *p* < .05.

## 3 RESULTS AND DISCUSSIONS

### 3.1 Weight loss and microbial population

#### 3.1.1 Weight loss

**Figure 1:**
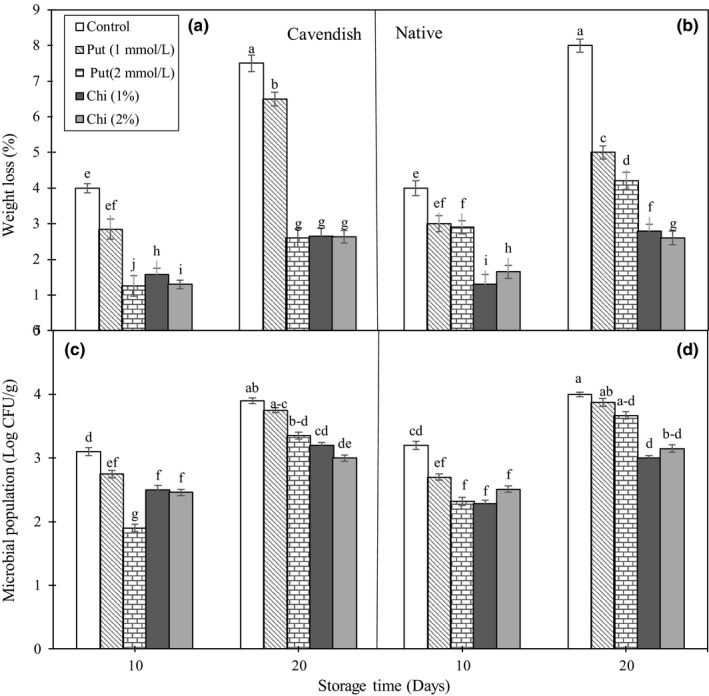
Effect of chitosan (1% and 2%) and putrescine (1 and 2 mmol/L) treatments on weight loss (%) (A and B) and microbial population (C and D) in fruits of “Native” and “Cavendish” cultivars after 0, 10, and 20 days. Control fruits were treated with distilled water. Means marked with the same letter are not significantly different (*p* < .05)

Fruit transpiration is expected to produce fruit weight losses during storage, and indeed, the control fruits of both cultivars lost weight gradually. Treatments with chitosan and putrescine had statistically significant (at *p* < .05) effects on weight loss. From 10 to 20 days, most of the treated fruits of both cultivars showed smaller weight losses compared to the control ones, with the only exception of the 1 mmol/L putrescine treatment at 10 days (Figure 1A and B). After 20 days, the treatments more effective were 1% and 2% chitosan, with the treatment with 2 mmol/L putrescine having a similar effect in “Cavendish” but not as good in “Native,” whereas 1 mmol/L putrescine was less effective in both cultivars. Chitosan treatments reduced weight loss by 65%–70% when compared to the controls.

Many climacteric fruits loss weight because of water evaporation from the skin surface during ripening and senescence (Sharma, Singh, & Goswami, 2001). The first weight loss mechanism in the harvested fresh fruits during storage includes diffusion of vapor between the internal and external phases by the vapor pressure gradient of the fruit juice (Suseno et al., 2014). In our case, application of chitosan was efficient in decreasing water loss from the fruit skin at both concentrations used, 1% and 2%, confirming previous results with 1.5 (Zahoorullah et al., 2017), 2 (Suseno et al., 2014), and 2.5% chitosan (Widodo et al., 2015).

#### 3.1.2 Microbial population

The effect of fungal pathogens such as *Penicillium expansum* is one of the major problems of banana during the postharvest period, along with physical damages during transportation. Results show that the microbial population in fruits increased gradually during storage in the control fruits (Figure 1C and D). After 20 days, most treatments decreased significantly microbial population when compared to the controls, with the only exceptions of the 1 mmol/L putrescine treatment in both cultivars and the 2 mmol/L putrescine treatment in “Native.” Therefore, treatments with chitosan were the most effective and provided similar results, whereas the two treatments with putrescine were less efficient, with the 2 mmol/L treatment giving better results than the 1 mmol/L one.

As an antifungal compound, chitosan can directly affect microorganisms and also affect indirectly the activity of peroxidase, chitinase, and glucanase enzymes, which increase the resistance of fruits against biological stresses (Zeng, Deng, Ming, & Deng, 2010). The antiputrescent and antimicrobial properties of chitosan are dependent on the type of chitosan, storage temperature, and chemical substances in the fruit (Devlieghere, Vermeulen, & Debevere, 2004). Previous studies have reported positive effects of chitosan on controlling putrescence in apple (Li et al., 2015) and strawberry, tomato, oranges, and banana (Sakif et al., 2016).

### 3.2 Total antioxidant activity, total phenolic compound concentration, and ascorbic acid (vitamin C) concentration

#### 3.2.1 Total antioxidant activity

**Figure 2:**
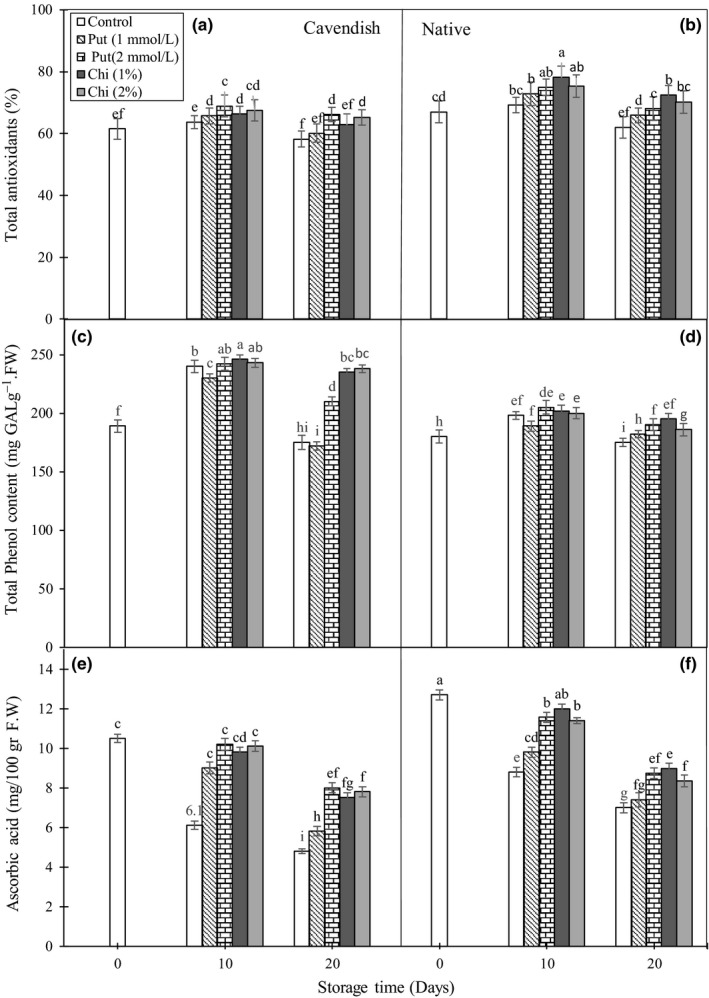
Effect of chitosan (1% and 2%) and putrescine (1 and 2 mmol/L) treatments on total antioxidants (A and B), total phenolic compound content (C and D), and ascorbic acid (E and F) in fruits of “Native” and “Cavendish” cultivars after 0, 10, and 20 days. Control fruits were treated with distilled water. Means marked with the same letter are not significantly different (*p* < .05)

The total contents of antioxidants in fruits are an important characteristic when considering their nutritional value. Total antioxidant activity in the controls was maintained at day 10 and decreased slightly by day 20. At day 20, all cover treatments led to similar slight increases in antioxidant activity when compared to the controls (Figure 2A and B).

In mango fruits, it has been reported that the antioxidant defense system and the reactive oxygen species (ROS) decrease and increase, respectively, at the beginning of fruit ripening and during senescence (Kim, Brecht, & Talcott, 2007). In the same crop, chitosan has been reported to increase the potential of ROS scavengers, leading to increased contents of phenolic compounds and antioxidants (Jongsri, Wangsomboondee, Rojsitthisak, & Seraypheap, 2016). Also, treatment with different concentrations of chitosan has also been reported to activate the antioxidant enzymes catalase (CAT), superoxide dismutase (SOD), and peroxidase (POD), which are an important part of the antioxidant potential during storage, in tomato (Liu, Tian, Meng, & Xu, 2007), orange (Zeng et al., 2010), and guava (Hong et al., 2012). On the other hand, polyamines play very important roles in the antioxidant system and in protecting plasma membrane phospholipids against ROS damages (Verma & Mishra, 2005). Applying different concentrations of polyamines such as putrescine has been reported to increase the activity of the antioxidant system in pomegranate (Mirdehghan et al., 2007; Razzaq et al., 2014) and plum (Davarynejad, Zarei, Ardakani, & Nasrabadi, 2013).

#### 3.2.2 Total phenolic compound concentration

The total concentration of phenolic compounds is an important component of the antioxidant capacity of fruits and provides an added value to the produce. During storage, the total phenolic compound contents increased in all fruits by day 10, to decrease afterward by day 20 (Figure 2C and D). At day 20, the decrease in phenolic contents was minimized by chitosan and putrescine treatments when compared to the control, with the only exception being the 1 mmol/L putrescine treatment in “Cavendish.” Generally speaking, the two chitosan treatments were the best in maintaining phenolic compounds in banana fruits, especially in “Cavendish.”

The decrease in phenolics in the flesh of the banana fruit during storage is thought to be related to increases in polyphenol oxidase activity (Sharma et al., 2001) and polymerization of tannins (Mirdehghan et al., 2007). Decreases in the activity of polyphenol oxidase after chitosan treatment have been reported for strawberry (Eshghi et al., 2014) and *Luffa cylindrica* (Han et al., 2014). Also, treatment with polyamines has been reported to lead to an increased amount of phenolics in mango (Razzaq et al., 2014) and pomegranate (Mirdehghan et al., 2007), in line with the results obtained here in the “Cavendish” cultivar of banana. The moderate decrease in phenolics in the putrescine treatments.. could be due to the important role of polyamines in delaying the development of polyphenol oxidase activity, associated with a decrease in fruit respiration. Polyamines have antioxidant activity, and a linear relationship has been found between antioxidant activity and phenolic compound contents in apricot and mango (Razzaq et al., 2014).

#### 3.2.3 Ascorbic acid concentration

Ascorbic acid in fruits is also an important component of the antioxidant capacity in fruits. In the controls, the concentration of ascorbic acid decreased by 35%–40% by day 10 and showed further decreases by day 20 (Figure 2E and F). All covering treatments decreased this loss of ascorbic acid, with the 2 mmol/L putrescine and both chitosan treatments being more effective. In these three treatments, the final loss of ascorbic acid after 20 days of storage was 25% and 30% in the cultivars “Cavendish” and “Native,” respectively, compared to losses of 55% and 45%, respectively, in the controls.

The ascorbic acid content in fruits increases in the ripening period and decreases during storage due to the activity of ascorbic acid oxidase. Coverings such as chitosan increase the activity of cytochrome oxidase by decreasing the internal O_2_ in fruits, and this enzyme can decrease significantly the decomposition rate of ascorbic acid (Özden and Bayindirli, 2002). The application of 1% and 2% chitosan has been previously reported to increase the ascorbic acid content in fruits of mango (Jongsri et al., 2016) and banana (Suseno et al., 2014), and in *L. cylindrica* (Han et al., 2014). Also, treatments with 2 mmol/L putrescine have been reported to prevent oxidation of ascorbic acid by decreasing water loss and maintaining the stability of the cell membranes (Mirdehghan et al., 2007). Numerous antioxidant properties have been reported for polyamines in different studies (Jimenez et al., 2002; Martínez‐Romero et al., 2000).

### 3.3 Fruit firmness and polygalacturonase enzyme activity

#### 3.3.1 Fruit firmness

**Figure 3:**
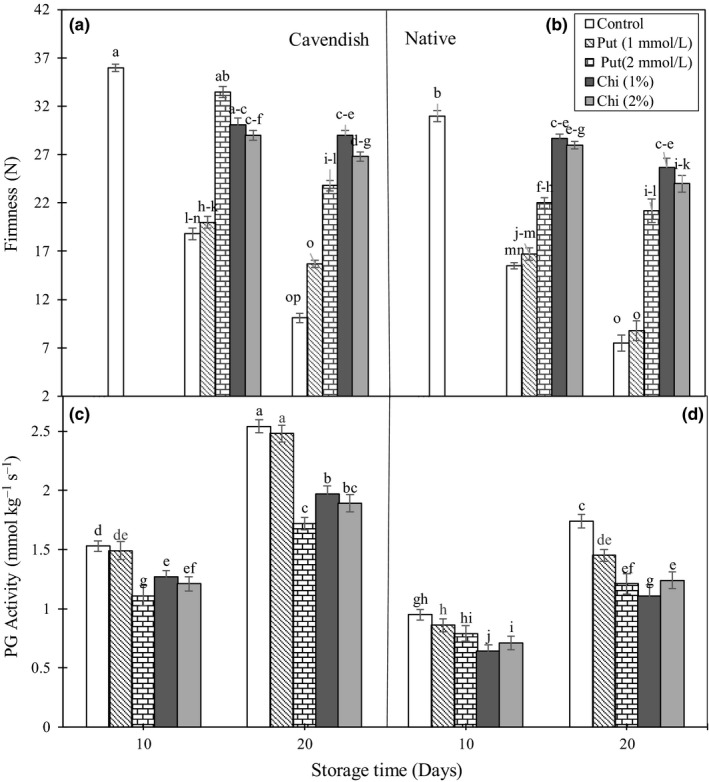
Effect of chitosan (1% and 2%) and putrescine (1 and 2 mmol/L) treatments on firmness (A and B) and PG activity (C and D) in fruits of “Native” and “Cavendish” cultivars after 0, 10, and 20 days. Control fruits were treated with distilled water. Means marked with the same letter are not significantly different (*p* < .05). PG, polygalacturonase

Firmness is a main feature regarding fruit quality. Banana is a soft fruit that suffers a rapid loss of firmness during ripening, and this contributes greatly to its short postharvest life. Results show that fruit firmness decreased markedly during storage in the controls of both cultivars, with 1 mmol/L putrescine having little effect (Figure 3A and B). However, banana fruits of both cultivars treated with 1% and 2% chitosan had much better firmness at day 20 when compared to the controls, with the 2 mmol/L putrescine treatment having a smaller effect.

Firmness of the fruit tissues is related to the structure of the cell walls. Alterations in the structure of this cell compartment, including decreases in hemicellulose and galactose, dissolution of pectins, the activity of hydrolyzing enzymes such as PG, and a rapid production of ROS soften fruit tissues during storage (Cheng et al., 2008). Any factor preventing or weakening the activity of enzymes decomposing the cell wall would preserve the firmness of fruit tissues. In previous works, chitosan has been found to maintain fruit tissue firmness in mango (Jongsri et al., 2016), and banana (Purwoko et al., 2002; Widodo et al., 2015; Zahoorullah et al., 2017).

#### 3.3.2 Polygalacturonase enzyme

Polygalacturonase is the major enzyme responsible for pectin disassembly in ripening fruits, and differences in firmness with tissue position in banana fruits have been shown to be positively correlated with variations in PG and/or cellulase activity (Gayathri & Nair, 2014). The PG activity increased markedly in control during storage (Figure 3C and D). After 20 days of storage, treatment with 1 mmol/L putrescine did not have any effect in “Cavendish” and only a minor effect in “Native.” However, treatment with 2 mmol/L putrescine, 1% chitosan, and 2% chitosan caused significant decreases in PG activity in both cultivars.

The application of putrescine at different concentrations has been found to cause decreases in the activity of cell wall‐decomposing enzymes, including exo‐PG, endo‐PG, and polyesterase (Razzaq et al., 2014). Banana always becomes vulnerable after being harvested, and this is particularly important in developing countries, where storage, transportation, and marketing equipment are often limiting factors. Therefore, the application of coverings that reduce quality loss and postharvest side effects and maintain physical properties will be beneficial in these cases.

### 3.4 TSS and TA of the fruits

**Figure 4:**
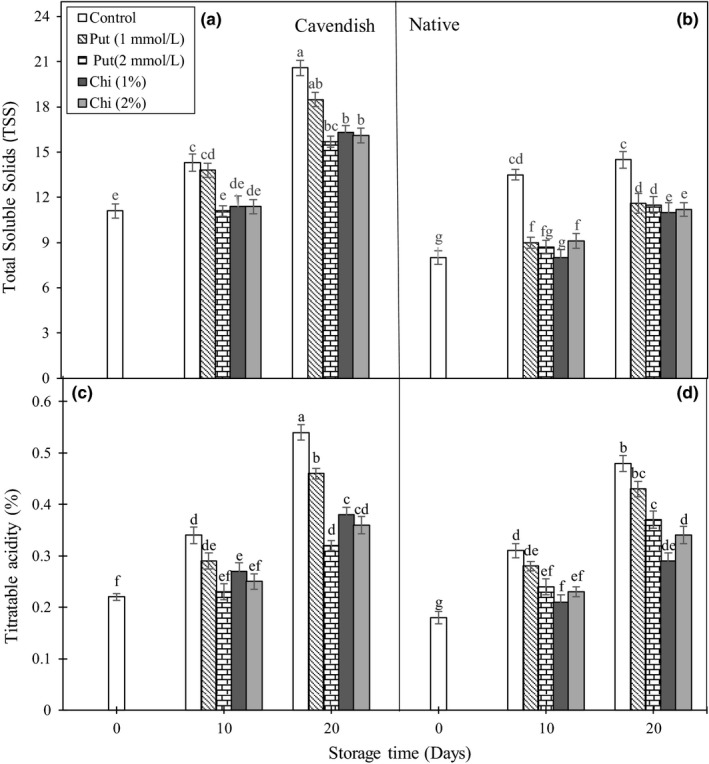
Effect of chitosan (1% and 2%) and putrescine (1 and 2 mmol/L) treatments on TSS (A and B) and TA (%) (C and D) in fruits of “Native” and “Cavendish” cultivars after 0, 10, and 20 days. Control fruits were treated with distilled water. Means marked with the same letter are not significantly different (*p* < .05). TSS, total soluble solids; TA, titratable acidity

The chemical composition of the fruit tissue is an important criterion when assessing fruit quality. Control banana fruits showed increases in TSS and TA during ripening (Figure 4A–D). After 20 days of storage, the 1 mmol/L putrescine treatment gave values similar to or close to the control ones for TA in both cultivars and TSS in “Cavendish,” whereas TSS were slightly decreased in “Native.” The treatments with 1% and 2% chitosan and 2 mmol/L putrescine gave TSS and TA values lower than those found in the controls, indicating that the changes occurring in the fruit during maturation and ripening are delayed.

Increases in fruit TSS can be related to several factors, including decomposition of starch into sugar (Arthey, 2005), reduced respiration rate and transformation of sugar into carbon dioxide and water (Eshghi et al., 2014), hydrolysis of cell wall polysaccharides (Comabella & Lara, 2013), and increased percentage of dry matter due to water loss (Dong, Cheng, Tan, Zheng, & Jiang, 2004). The decreases in fruit TSS with the treatments can also be associated with changes in the fruit internal atmosphere of the fruit, including decreases in O_2_ and ethylene and increases in CO_2_ (Devlieghere et al., 2004), which would result in decreases in respiration and conversion of starch into sugar. Chitosan applications have been reported to cause decreases in fruit TSS and TA during storage in papaya (Ali et al., 2011) and banana (Suseno et al., 2014; Zahoorullah et al., 2017), and to decrease significantly the acid content in strawberry (Petriccione et al., 2015), litchi (Dong et al., 2004), and banana (Widodo et al., 2015; Zahoorullah et al., 2017). On the other hand, application of polyamines has been reported to delay the increases in TSS during ripening in mango (Malik & Singh, 2005) and grape (Mirdehghan and Rahimi, 2016), and application of putrescine decreased TSS in pomegranate (Razzaq et al., 2014).

### 3.5 Ethylene

**Figure 5:**
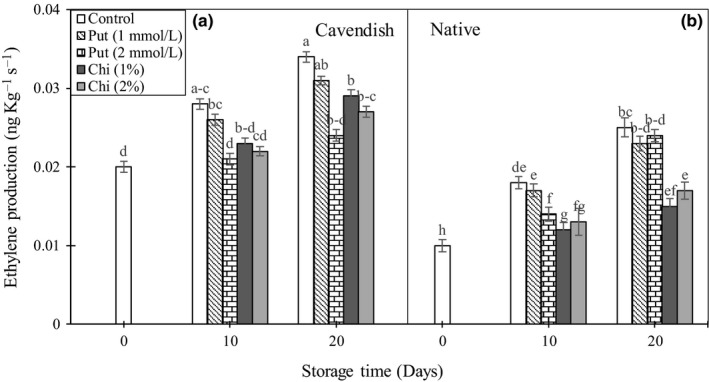
Effect of chitosan (1% and 2%) and putrescine (1 and 2 mmol/L) treatments on ethylene production (A and B) in fruits of “Native” and “Cavendish” cultivars after 0, 10, and 20 days. Control fruits were treated with distilled water. Means marked with the same letter are not significantly different (*p* < .05)

Ethylene is a natural senescence hormone which is produced by plant tissues at varying rates. Ethylene production increased gradually during storage in control banana fruits, whereas application of 1 mmol/L putrescine did not cause any effect (Figure 5A and B). After 20 days of storage, both chitosan treatments reduced ethylene production in the two banana cultivars, whereas the 2 mmol/L putrescine decreased ethylene production only in “Cavendish.”

Previous works have shown that chitosan, acting as a barrier film, provided an altered internal atmosphere and a selective membrane for permeation of ethylene in and out of the fruit and decreased production of ethylene by the fruit (Ali et al., 2011). Similar results have been reported for fruits such as mango (Jongsri et al., 2016), papaya (Ali et al., 2011), and litchi (Dong et al., 2004). On the other hand, preharvest application of polyamines in tomato (Li et al., 2005) and banana (Purwoko et al., 2002) decreased significantly the activity of ACC oxidase, a key enzyme in the ethylene production pathway, and spermidine and putrescine were shown to delay fruit ripening by decreasing respiration and ethylene production (Malik & Singh, 2005).

### 3.6 Color luminescence (L*)

**Figure 6:**
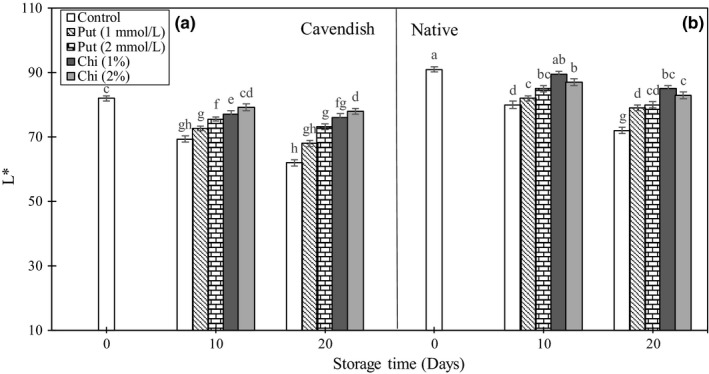
Effect of chitosan (1% and 2%) and putrescine (1 and 2 mmol/L) treatments on L* (A and B) in fruits of “Native” and “Cavendish” cultivars after 0, 10, and 20 days. Control fruits were treated with distilled water. Means marked with the same letter are not significantly different (*p* < .05)

Lightness of the fruit peel is an important selection criterion for the consumer, as it gives an indication of the state of fruit ripening. In the control fruits, there was a progressive decrease in fruit lightness, assessed using the L* value, during storage (Figure 6A and B). After 20 days of storage, most treatments led to increases in L* when compared to the controls, with the exception of the 1 mmol/L putrescine treatment in “Cavendish.” Treatments with chitosan and 2 mmol/L putrescine generally provide the best results regarding the maintenance of fruit lightness.

Browning of banana is one of the most important damages during storage, which decreases the quality, shelf‐life, and marketability of the product. The main reason of browning is the polyphenol oxidase enzyme, which causes oxidation of the phenols by destroying the cell wall and creating dark and brown colors (Sharma et al., 2001). It has been reported that application of chitosan on strawberry (Petriccione et al., 2015) and polyamines on grapes (Mirdehghan and Rahimi, 2016) and banana (Purwoko et al., 2002) delayed the decreases in L*.

## 4 CONCLUDING REMARKS

Banana is a climacteric fruit which is generally harvested at ripe green stage, and when ripening of the fruit begins, chlorophyll starts to decompose, carotenoids appear in the fruit peel, and respiration and ethylene production increase. Changes in the physical characteristics of the control fruits after 20 days of storage shown in this work include a significant weight loss (7%–8%), major decreases in firmness (70%–75%), and marked decreases in lightness (20%–25%). In the same period, there are profound changes in the fruit chemical and biochemical characteristics, including small decreases in antioxidants (5%–7%) and phenolics (3%–8%), marked decreases in ascorbic acid (45%–55%), marked increases in PG activity (66%–83%) and TSS (80%–90%), and major increases in TA (145%–167%) and ethylene (30%–120%). Chitosan treatments (1%–2%) protect banana fruits to a great extent, as changes in fruit physical characteristics are diminished, with weight losses and decreases in firmness and lightness being reduced to 3%–4%, 30%–35%, and 5%–9%, respectively. Also, chitosan treatments diminish changes in fruit chemical characteristics, including smaller decreases in ascorbic acid (25%–35%) and smaller increases in PG activity (25%–30%), TSS (40%–46%), TA (65%–90%), and ethylene (35%–70%); in fruits treated with chitosan, there were even minor increases in antioxidants (5%–7%) and total phenolic compounds (3%–26%). On the other hand, putrescine (at 1 and 2 mmol/L) was less effective than 1%–2% chitosan in protecting fruits. As the effects of 1% and 2% chitosan were quite similar, further studies can be focused on the effects of even lower concentrations of chitosan, which could be beneficial for fruit companies because of the reduced cost of the treatments (in previous studies with banana, the chitosan range used was 0.5%–2.5%; Sakif et al., 2016; Suseno et al., 2014; Widodo et al., 2015; Zahoorullah et al., 2017). Finally, it should be stated that the experiments presented here were carried out with chemically pure compounds, resulting in costs of 0.2 and 13 euros per L of solution for 1 mmol/L putrescine and 1% chitosan, respectively. Of course, much lower prices can be found for bulk grade chitosan, reducing 100 times the cost of the treatment, although the effectiveness of any specific bulk grade chitosan should be tested.

## CONFLICT OF INTEREST

The authors declare no conflict of interest.
